# Supplementary material for: Click Decoration of Bombyx mori Silk Fibroin for Cell Adhesion Control
Source: Molecules. 2020 Sep 8;25(18):4106. doi: 10.3390/molecules25184106 (PMC7570510; doi:10.3390/molecules25184106)
Supplement: Supplementary file 1 [file molecules-25-04106-s001.pdf]

-Supporting Information-

Click Decoration of *Bombyx mori* Silk Fibroin for Cell Adhesion Control

Hidetoshi Teramoto [1,*], Minori Shirakawa [2] and Yasushi Tamada [2,*]

[1] Silk Materials Research Unit, Division of Biotechnology, National Agriculture and Food Research Organization (NARO), 1-2 Owashi, Tsukuba, Ibaraki 305-8634, Japan
[2] Faculty of Textile Science and Technology, Shinshu University, 3-15-1 Tokida, Ueda, Nagano 386-8567, Japan

Additional Methods

Click modification test

Aliquots of alkaline-degummed cocoon (fibroin) were dissolved in 8 M LiBr aqueous solution at 35°C at a concentration of 50 μg/μL. The dissolved fibroin was reacted overnight with carboxyrhodamine 110 DBCO at room temperature. The reaction condition was as follows: 4 μg/μL fibroin, 0.1 M carboxyrhodamine 110 DBCO, 0.64 M LiBr, 50 mM Tris-HCl (pH 8), 6.56 M urea, and 5% (v/v) DMSO. After the reaction, 15 μL each of the reaction solutions were mixed with 5 μL 8 M urea and 10 μL 6×sample buffer (Nacalai Tesque, Kyoto, Japan), and were then incubated for 15 min at 50°C. The mixtures were separated by electrophoresis on a 4-15% Mini-PROTEAN TGX Precast Gel (Bio-Rad, Hercules, CA, USA) at 100 V. Fluorescence of the gel was observed with a Fusion FX7 chemiluminescence imaging system (Vilber-Lourmat, Marne-la-Vallée, France). The gel was stained with Bio-Safe Coomassie Stain (Bio-Rad) followed by destaining with deionized water. The CBB-stained gel was observed with a Fusion FX7 chemiluminescence imaging system.

Contact angle measurement

To the fibroin-coated 60 mm dish, 1.5 mL/well of DBCO-mPEG (0 and 300 μM) of 20 kDa chain length dissolved in 50 mM Tris-HCl (pH 8) / 50% (v/v) DMSO was added. The dishes were incubated overnight at 50°C. After removing the DBCO-mPEG solution, the wells were washed with 50% (v/v) DMSO and deionized water, and were then dried at room temperature. The bottoms of the dishes were cut into four pieces. Contact angles of deionized water on three different points of each piece were measured using a FTA188 video tensiometer (First Ten Ångstroms, Newark, CA, USA). Samples from three independent experiments were used for the measurements.

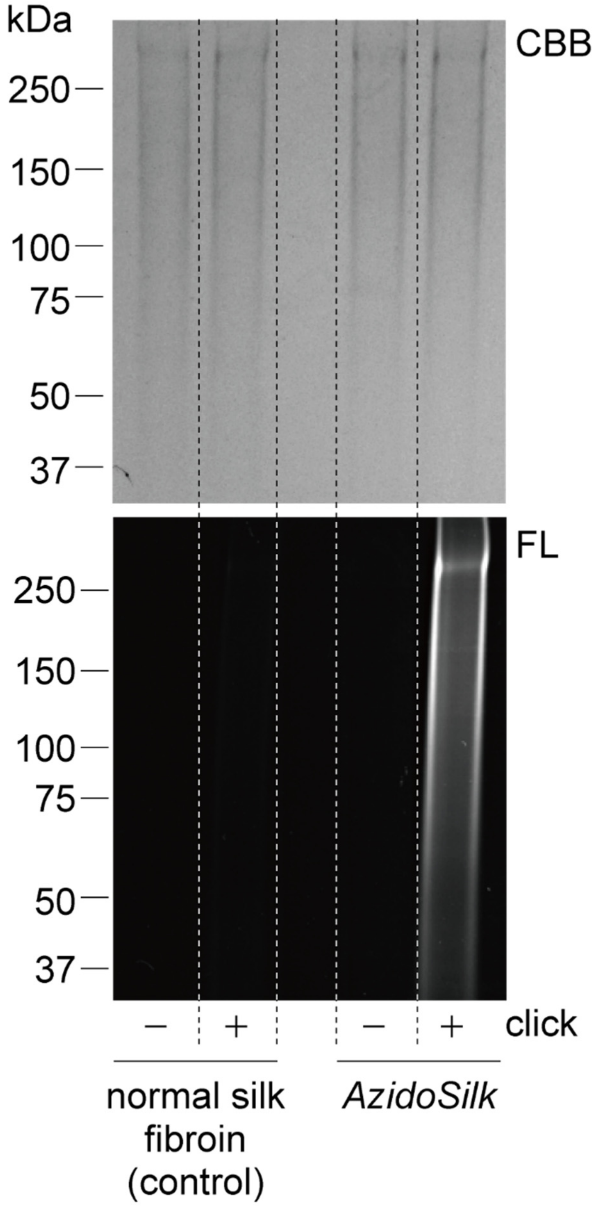

Figure S1. Click modification tests of normal silk fibroin and *AzidoSilk* with a fluorescent probe. Fibroin samples after the click reaction with carboxyrhodamine 110 DBCO were separated by SDS-PAGE and their fluorescence was recorded (bottom panel). The same gel was stained with CBB (upper panel).

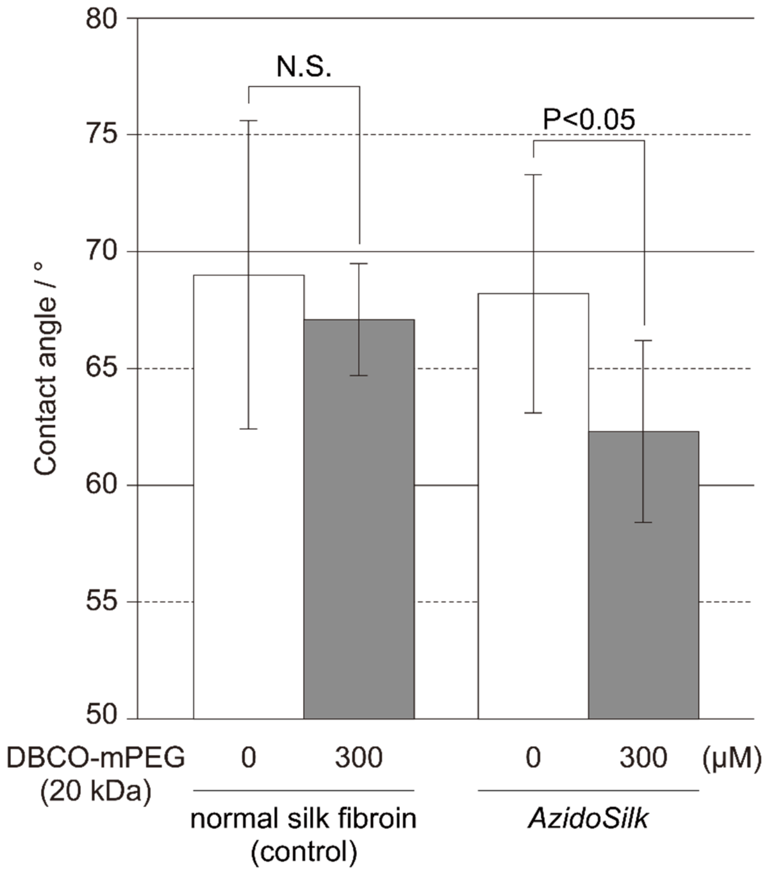

Figure S2. Contact angles of normal silk fibroin and *AzidoSilk* treated with DBCO-mPEG of 20 kDa chain length. The error bars represent standard deviation (n = 12). Statistical significance by the Tukey-Kramer test is shown. N.S. denotes no statistical significance ($P>0.05$).

A

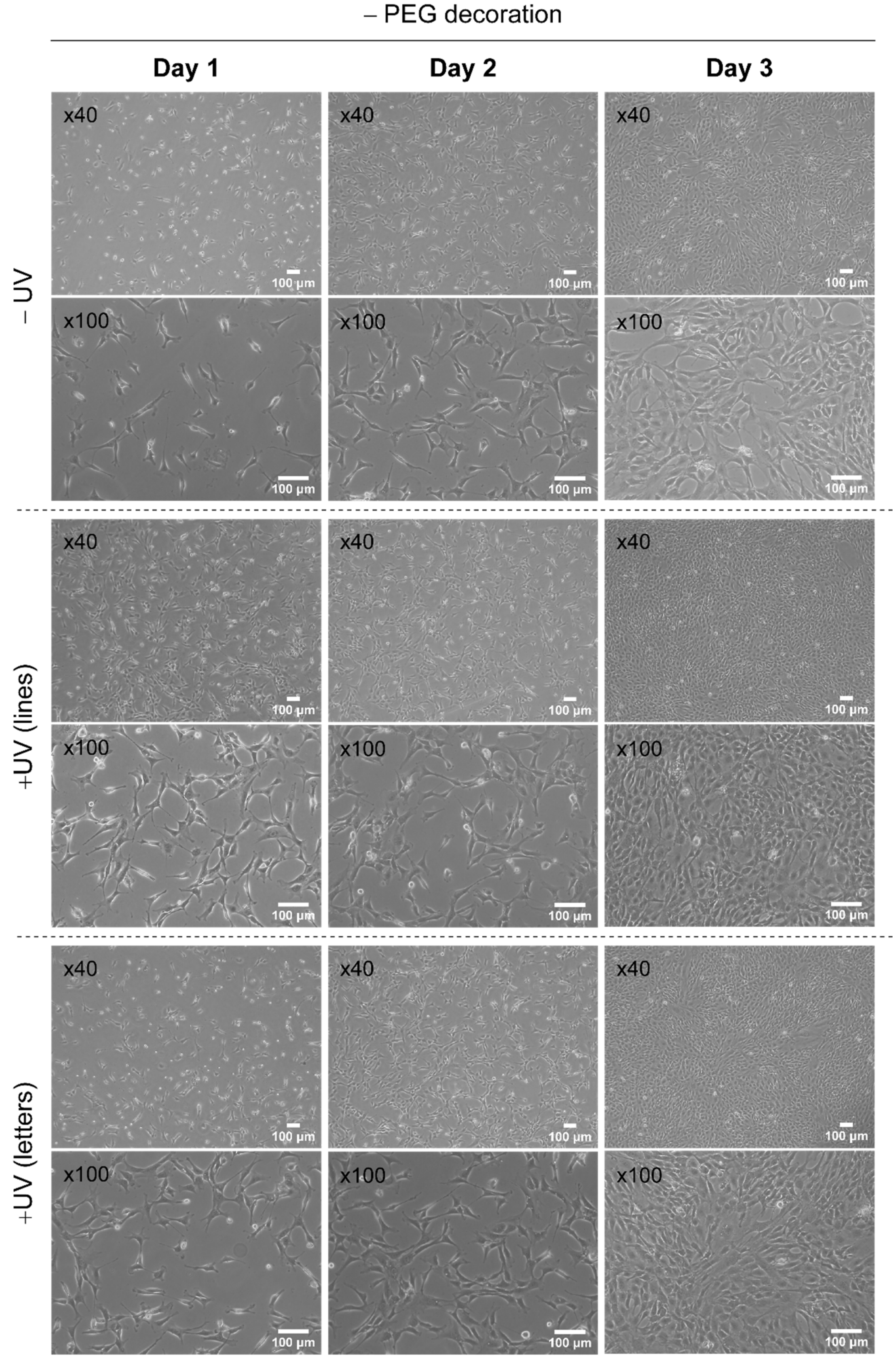

– PEG decoration
Day 1
Day 2
Day 3
– UV
+UV (lines)
+UV (letters)
x40
x100
100 μm

(continued)

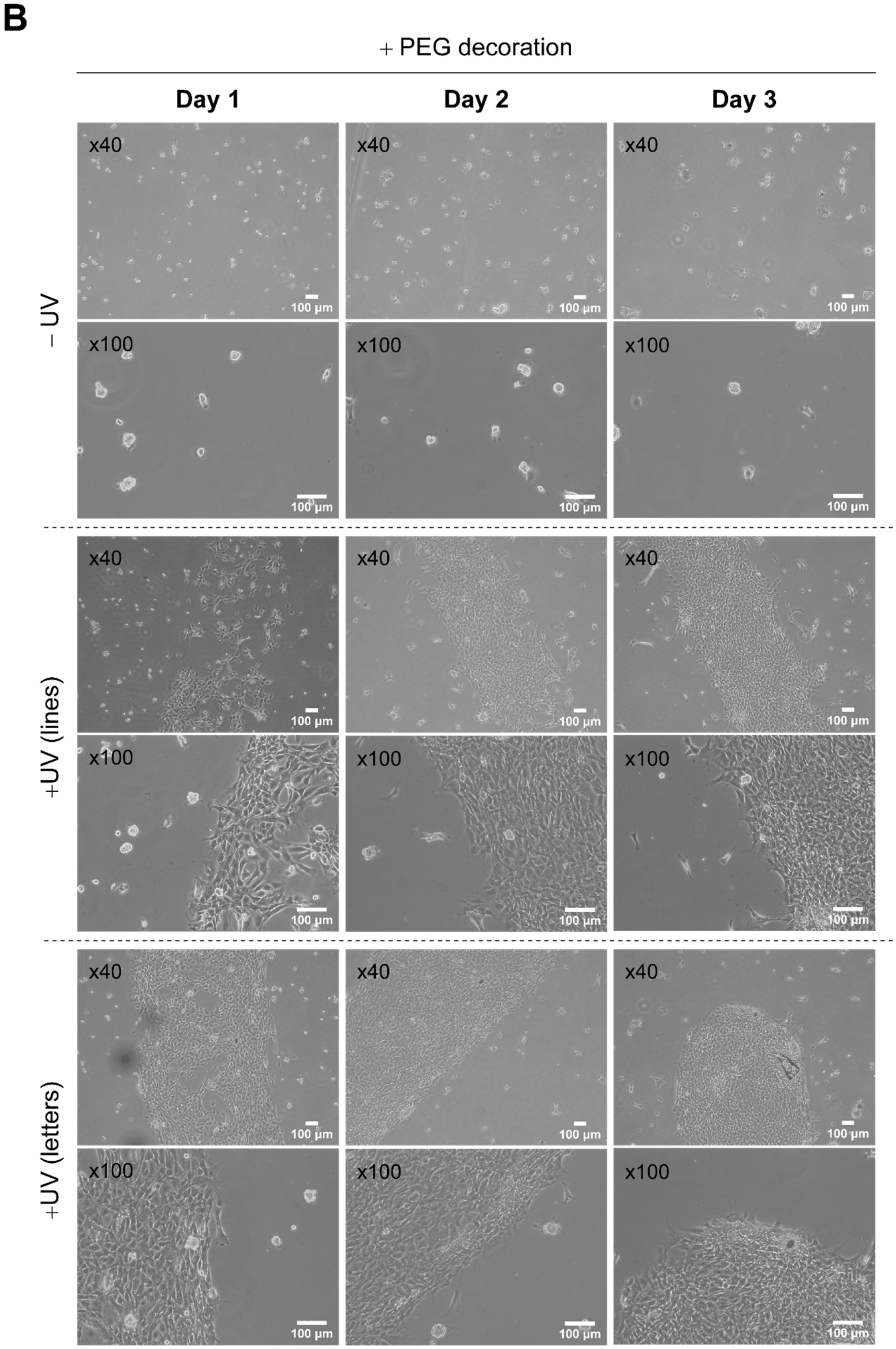
B
+ PEG decoration
Day 1
Day 2
Day 3
– UV
+UV (lines)
+UV (letters)
x40
x100
100 μm

(continued)

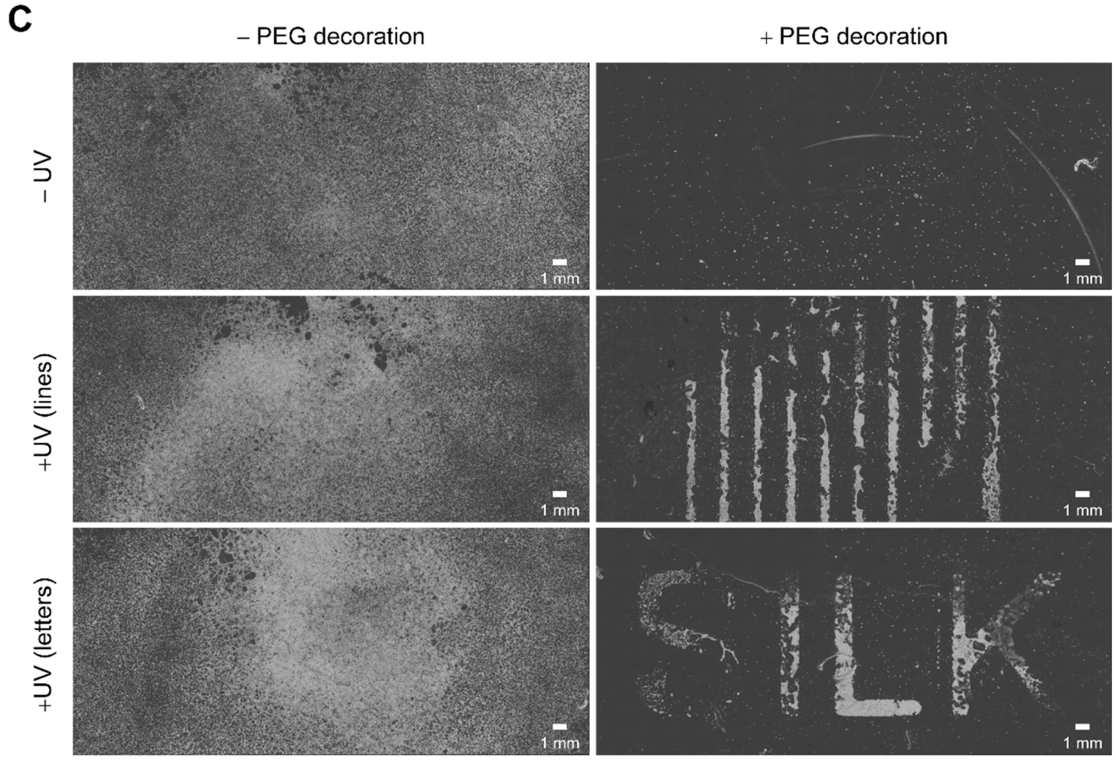

Figure S3. Spatial patterning of NIH3T3 fibroblasts on *AzidoSilk* film modified with DBCO-mPEG of 20 kDa chain length after partial irradiation with 254 nm UV light through photomasks with line (Figure 5A) and alphabetical (Figure 5D) patterns. Fibroblasts were cultured for 3 days in Eagle MEM supplemented with 10% FBS. (A) Cell proliferation without PEG decoration. Scale bars are 100 μm. (B) Cell proliferation with PEG decoration. Scale bars are 100 μm. (C) Wide views after cell fixation by 100% methanol followed by drying. Cells turned into white after fixation. Scale bars are 1 mm.
